# Enhancement Effect of Aggregates on the Low-Temperature Cracking Resistance of Asphalt Mixtures

**DOI:** 10.3390/ma17122865

**Published:** 2024-06-12

**Authors:** Jianhuan Du, Zhu Fu

**Affiliations:** School of Architecture and Civil Engineering, Chengdu University, Chengluo Avenue No. 2025, Chengdu 610106, China; fuzhu@stu.cdu.edu.cn

**Keywords:** road engineering, asphalt mixture, indirect tensile test, XFEM, aggregate enhancement, crack configuration force

## Abstract

Aggregates’ configurations result in different stress fields, which change the fracture mode and mechanical properties of an asphalt mixture. To reveal the enhancing effect of aggregates with different particle sizes on the low-temperature cracking resistance of an asphalt mixture, an indirect tensile (IDT) test was carried out to analyze the aggregates’ influence on crack propagation and low-temperature cracking resistance from a macroscopic perspective. And combined with the test results, mesostructure models of an asphalt mixture with different aggregates’ spatial distributions were established through the extended finite element method (XFEM) to analyze changes in the crack propagation path and crack tip configuration force from a mesoscopic perspective. The main results showed that the crack tip configurational force was reduced due to the aggregate size increasing, demonstrating the inhibitory effect of aggregates on crack propagation. This contributes to enhancing asphalt mixtures’ low-temperature cracking resistance. Compared to single-grain aggregates, multi-grain aggregates exhibit a greater inhibitory effect on crack propagation. Nonetheless, an excessive disparity in particle sizes compromises particle continuity, leading to the formation of more branching cracks. Meanwhile, the aggregates’ inhibitory effect on crack propagation is influenced by the crack deflection angle. In particular, when the crack deflection angle, *β*, equals 45°, the crack tip’s configurational force is notably larger, leading the crack to enter an unstable state conducive to the expansion and formation of macrocracks. The research results reveal aggregates’ inhibitory effect on crack propagation from a macro- and microperspective and reveal the relationship between aggregate configurations and the low-temperature cracking resistance of asphalt mixtures.

## 1. Introduction

At the mesoscale, asphalt mixtures are seen as multiphase mixtures composed of aggregates and asphalt mortars [1,2,3,4]. Macroscopic tests, such as the composite load fracture test [5,6,7,8], the semi-circular bend (SCB) test [9,10], and the trabecular fracture test [11,12], illustrate that the properties of an asphalt mixture’s components directly affect its low-temperature cracking resistance. Many conventional tests have been carried out to analyze the various influential factors’ effects on crack propagation and asphalt mixtures’ fracture toughness. These influencing factors include mechanical properties [13], fiber content [14,15], recycled asphalt pavement (RAP) content [16], deicer materials [17], and the presence of asphalt modifiers [17].

The novel edge-notched disc bend (ENDB) test [18] was proposed to facilitate studies related to the effect of void fraction and temperature variation on the fracture behavior of hot mix asphalt (HMA). The influence of factors such as temperature, loading rate, and fiber content on the fracture behavior of asphalt mixtures has been studied by using the ENDB test [19]. However, asphalt mixtures’ macroscopic mechanical behavior is also directly affected by aggregates’ natural mechanical properties, gradation, configuration, roundness, and spatial distribution [20,21,22,23,24].

The effect of aggregate type, size, and distribution on the fracture behavior of pavement has been investigated by customizing a model of material properties [25]. Asphalt mixtures’ fracture toughness values were obtained through the UGR-FACT fracture test and SCB test, revealing the relationship between aggregates’ mechanical properties and crack propagation [26,27]. The geometric characteristics of aggregates have been scanned by digital image processing (DIP) methods to analyze their spatial distribution characteristics, including their aggregate shape index [28] and aggregate roundness [29]. Wang [30] used DIP to analyze the effects of aggregate structure on the mechanical properties of asphalt mixtures by quantifying the distribution and orientation of aggregates in space as well as the mutual contact properties between aggregates. Furthermore, the accelerated polishing test and X-ray diffraction were carried out to establish a mathematical model that is better suited to determining the proportion of steel slag to other aggregates [31].

In addition, configurations of aggregates, voids, and cracks change, resulting in changes in the free energy of materials, so the configurational force is determined to describe the free energy changes caused by configurational changes in the materials [32,33]. Additionally, the maximum equivalent configurational force is proposed as the fracture criterion [34]. Based on the materials’ configurational mechanics (MCMs) [35,36], the extended finite element method (XFEM) is used to reveal the relationship between the energy dissipation and configurational force in different materials, such as brittle materials [37], viscoelastic and elastoplastic materials [38,39], and superelastic materials [40], respectively. Moreover, the interaction between I mode crack and inclusions [41,42] and the shielding effect of the II mode crack tip plastic zone [43] were also revealed through changes in the configurational force. And the SCB test results pointed out the influence of crack location on the crack resistance of asphalt pavements of different types [44].

In summary, previous studies have focused on revealing the influence of the geometric characteristics of aggregates on the fracture behavior and cracking resistance of asphalt mixtures using both traditional tests and image processing techniques. However, different aggregates’ configurations result in different stress fields, which change the fracture mode and mechanical properties of an asphalt mixture. In this study, asphalt mixture specimens were prepared to analyze the enhancement effect of aggregates through an indirect tensile (IDT) test. And pre-cracked asphalt mixture specimens were manufactured using waterjet cutting technology to investigate aggregates’ enhancement effect on the crack resistance of asphalt mixtures with different crack configurations. Moreover, based on the MCMs, the crack propagation process was numerically implemented through the XFEM to analyze the interference effects of aggregates with different configurations on crack propagation behavior from macroscale and mesoscale perspectives, such as crack resistance, the crack propagation path, and the crack tip’s configurational force.

## 2. Materials and Methods

### 2.1. Materials

At the mesoscale, asphalt mixtures are multiphase composite materials in which asphalt mortar is the matrix and coarse aggregates (particle size *D* > 2.36 mm) are regarded as enhancement materials. Asphalt mortar and asphalt mixtures with different aggregate sizes were designed based on their ratios in a dense AC-13 asphalt mixture [45,46], as shown in Table 1 and Table 2. Previously, volume index labratory test results [45] indicated that the coarse aggregate content is 57.5%, with an asphalt mortar oil-to-stone ratio of 12.7%. The best asphalt dosage in this work was 11.3%. And the asphalt adopts PPA-SBS (polyphosphoric acid and styrene butadiene styrene, Produced in Chengdu, Sichuan Province, China) composite-modified asphalt; its performance grade (PG) is PG 70-28, and the SBS and PPA contents are 3% and 1%, respectively. 

### 2.2. Indirect Tensile Test

**Figure 1:**
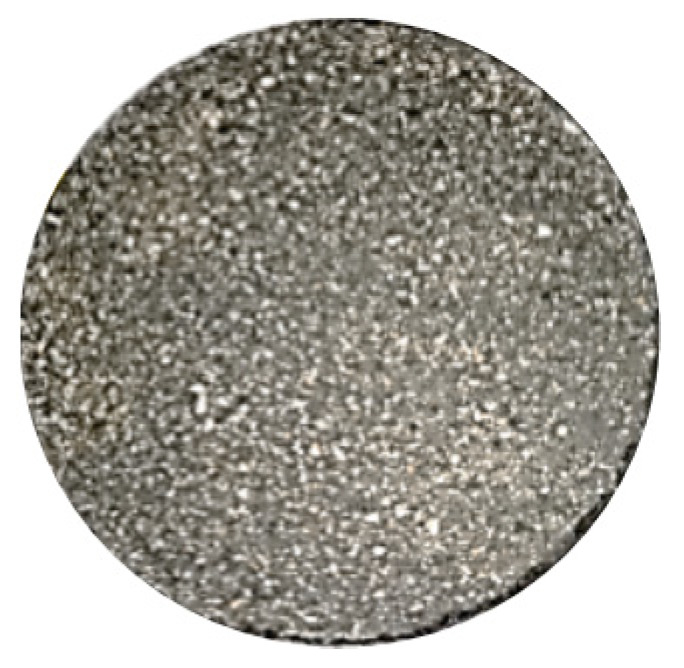
Asphalt mortar specimen.

A cylindrical asphalt mixture test specimen with a size, Φ, of 101.5 mm× (63.5 mm ± 1.3 mm) was prepared using the static pressure method (see Figure 1).

**Figure 2:**
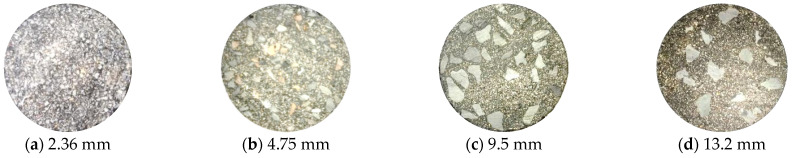
Asphalt mixture specimens containing single-grain aggregates.

**Figure 3:**
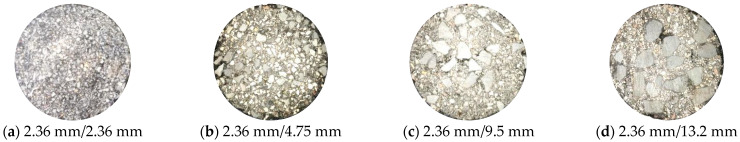
Asphalt mixture specimens containing multi-grain aggregates.

The same preparation and molding methods were used to prepare asphalt mixture specimens with multiple coarse aggregate sizes. The corresponding mass of coarse aggregate was added to the fully mixed asphalt mortar, as shown in Figure 2 and Figure 3.

The IDT test was carried out at −20 °C (±1 °C), and the loading model was a constant displacement loading. Furthermore, to avoid rapid crack propagation resulting from the high loading rate, the loading rate was set to 1 mm/min.

### 2.3. Extended Finite Element Method (XFEM)

**Figure 4:**
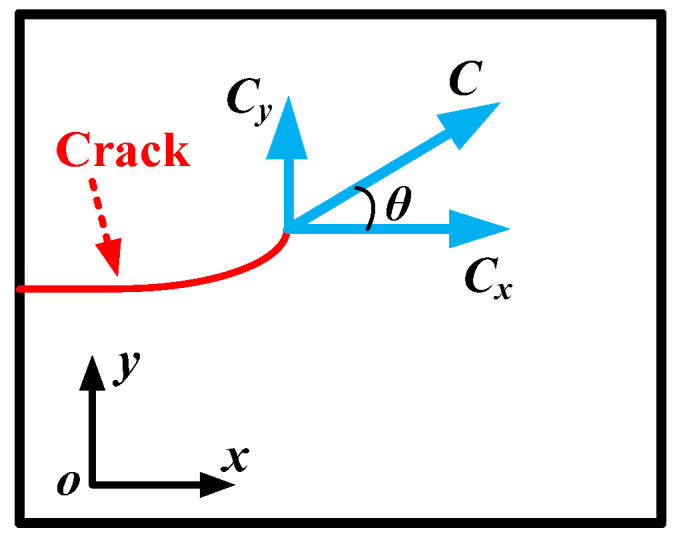
The configurational force fracture criterion.

The non-symmetric material structure or mixed-mode loading causes deviation in the crack propagation path. And based on the MCM, a theory is proposed to predict the crack propagation behavior, as shown in Figure 4. 

According to the *C*-force criterion, two basic stipulations on crack propagation are made in plane problems:

(1) If the configurational force, *C*, overcomes the material resistance, a crack begins to propagate, i.e.,

(1) $$\left|C\right|=\sqrt{C_{x}^{2}+C_{y}^{2}}\geq C_{\mathrm{th}}$$

where *C*_th_ is a material constant related to the material’s fracture resistance, which is independent of crack configurations and loading conditions. |*C*| is the configurational force associated with the material properties and stress intensity factors, and its component expression in the x-direction is shown as follows:

(2) $$C_{x}=\frac{K_{I}^{2}+K_{II}^{2}}{E}+\frac{1+v}{E}K_{III}^{2}$$

where *E* and *ν* are the elasticity modulus and poisson ratio, respectively, and *K_I_*, *K_II_*, and *K_III_* are the stress intensity factors for the I mode, II mode, and III mode, respectively.

(2) The crack propagates in the direction ahead of the crack tip along the configurational resultant force, *C*. The crack propagation angle, *θ* (see Figure 2), can be determined by

(3) $$\theta =\arctan\left(\frac{C_{y}}{C_{x}}\right)$$

Therefore, by combining this with Equations (2) and (3), the crack tip configurational force can be calculated.

**Figure 5:**
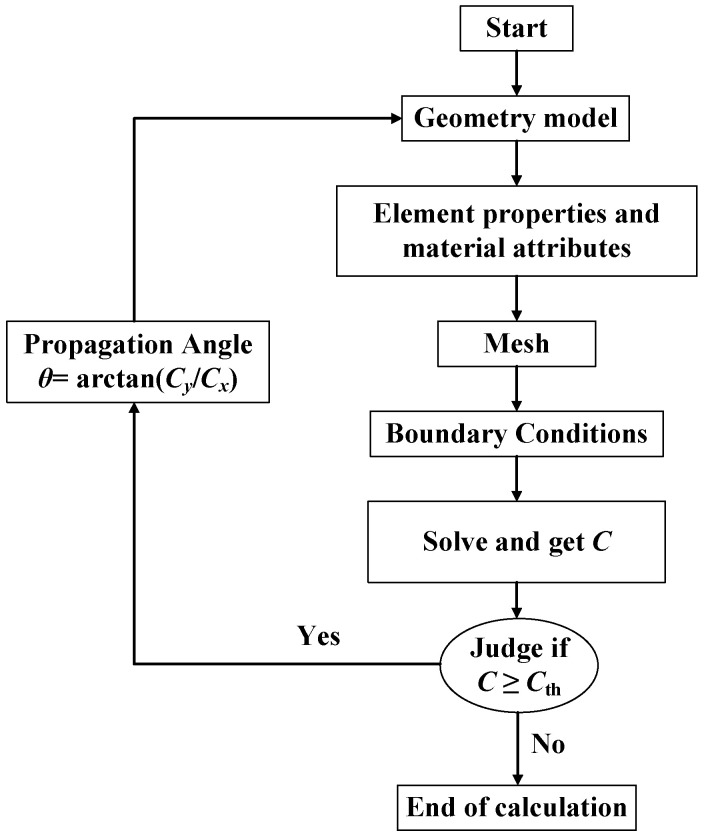
Numerical simulation process of crack propagation in ABAQUS.

If *C* ≥ *C*_th_, the crack continues to propagate, the crack propagation angle, *θ*, can be calculated from Equation (3), and the extended crack tip is taken as the new crack tip (see Figure 5); if *C* < *C*_th_, the crack does not propagate, and the calculation stops.

The basalt crushed stone is taken as the aggregate, and its elastic modulus, Poisson’s ratio, tensile strength, and internal friction coefficient are 55.5 GPa, 0.25, 27.6 MPa, and 0.5 [47,48], respectively. And the mechanical properties of the AC-13 gradation asphalt mixture at −20 °C are shown in Table 3.

### 2.4. Crack Propagation Path Extraction

In ABAQUS 6.14, the PHILSM variable is a signed distance function used to describe the crack surface. And the crack tip only stays on each element’s boundary, meaning the crack propagation path can be considered a straight line in each element. Therefore, the jump function, *H*(*x*), can be specified as the solution for the PHILSM variable to obtain the crack propagation path in each element.

**Figure 6:**
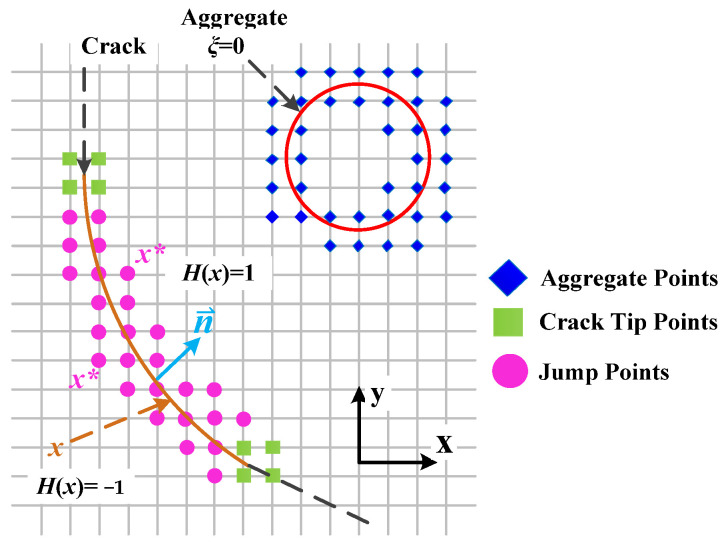
The jump function, *H*(*x*), schematic diagram.

The jump function, *H*(*x*), is described as follows:

(4) $$H(x)=\left\{\begin{array}{l} 1\;\;\;\;\;\;if\;(x-x*)n\geq 0\\ -1\;\;\;\;\mathrm{otherwise} \end{array}\right.$$

where *x* is a sample point on the crack surface; x* is the node of the element through which the crack passes, and ***n*** is the unit outer normal vector, as shown in Figure 6.

**Figure 7:**
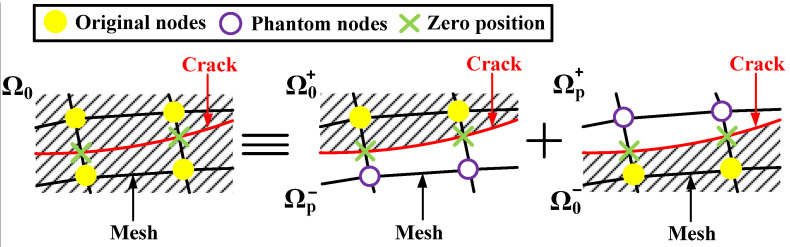
PHILSM field variables.

Moreover, the crack tip location on each element’s boundary can be obtained from the PHILSM zero position in each element, as shown in Figure 7. And the total crack length can be obtained by cumulatively summing up the crack length in each element.

## 3. Enhancement of Asphalt by Single-Grain Aggregates

### 3.1. Crack Propagation in Uncracked and Pre-Cracked Specimens

**Figure 8:**
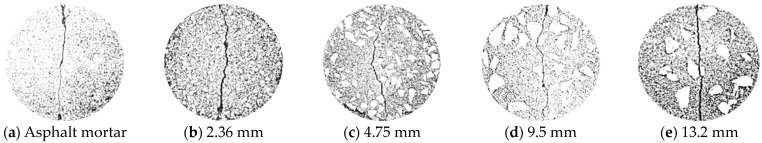
Images of single-grain aggregates specimens without pre-cracks.

To directly observe the internal crack propagation in the samples, test specimen images were binarized with digital image processing technology, as shown in Figure 8.

When the aggregate size, *D*, was below 4.75 mm (see Figure 8) for specimens without pre-cracks, the main crack propagated along a certain angle related to the load direction, which means that crack propagation significantly deviated due to the aggregates’ interference effect. Furthermore, increasing the aggregate size resulted in higher deviation. However, when the aggregate size, *D*, was larger than 4.75 mm, the crack expansion deviation was significantly reduced. It should be noted that the crack expansion deviations for the aggregate size *D* = 13.2 mm and the asphalt mortar exhibit similarities (see Figure 8). This is because the 13.2 mm aggregate did not significantly interfere with crack propagation, which is due to the lower amount of aggregate content and the loose spatial distribution of this test specimen. This analysis reveals that aggregates with a particle size of 4.75 mm most significantly interfered with the internal crack propagation of the asphalt specimens without pre-cracks. 

**Figure 9:**
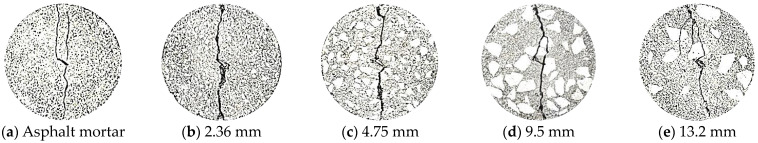
Images of single-grain aggregates specimens with pre-cracks.

The crack propagation results for the test specimens with pre-cracks (with a crack length of *l* = 10 mm and a crack deflection angle of *β* = 45°) are shown in Figure 9. The number of branching cracks increased with increasing aggregate size, indicating that larger aggregates had a weaker inhibitory effect on internal crack propagation.

Moreover, the number of branching cracks in the specimens without pre-cracks was lower than the number of branching cracks in the specimens with pre-cracks for aggregate particle sizes of *D* ≤ 4.75 mm. This means that the aggregates effectively inhibited crack growth within the asphalt material.

### 3.2. Aggregate Enhancement Effect

Low-temperature cracking resistance was used as an index to assess the test results, as shown in Table 4. The enhancing effect of the aggregate is expressed as the ratio of the low-temperature cracking resistance of the asphalt mixture with aggregate to the low-temperature cracking resistance of the asphalt mortar (without aggregate).

At −20 °C, the low-temperature cracking resistance of the asphalt mixture gradually increased with increasing aggregate particle size (see Table 4). Figure 4 and Figure 5 show that the asphalt mixture with pre-cracks had significantly reduced low-temperature cracking resistance compared to the mixture without pre-cracks. However, the enhancing effect of the aggregates was greater in the pre-cracked specimens because the aggregates effectively inhibited internal crack propagation.

**Figure 10:**
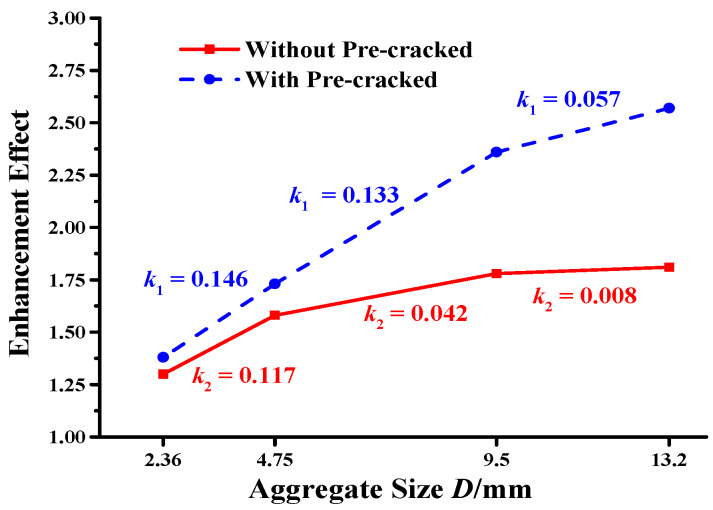
Aggregate enhancement effect with different aggregate sizes.

Figure 10 shows the relationship between aggregate enhancement and aggregate particle size. The slope, *k*, between two adjacent points in Figure 10 represents the enhancing effect of the aggregate on low-temperature cracking resistance. Slopes *k*_1_ and *k*_2_ represent the aggregate enhancement effect on the asphalt mixture with and without pre-cracks, respectively.

According to Table 3 and Figure 10, the aggregate enhancement effect gradually increased with increasing aggregate size, resulting in a corresponding gradual increase in the low-temperature cracking resistance of the asphalt mixture. However, the decline in *k* with increasing particle size shows that the aggregate enhancement effect, which increased with increasing particle size, did not necessarily increase as rapidly for larger particle sizes (i.e., diminishing returns). Moreover, under the same test conditions, different crack configurations led to different aggregate enhancing effects. For all aggregate sizes, slope *k*_1_ exceeded *k*_2_, showing that the aggregate reinforcement effect was enhanced for the pre-cracked samples.

## 4. Enhancement of Asphalt Mixture by Multi-Grain Aggregates

### 4.1. Crack Propagation in Uncracked Specimens

**Figure 11:**
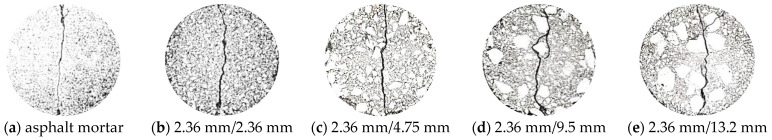
Images of multi-grain aggregates specimens without pre-cracks.

To directly observe the crack propagation path of the samples, the test specimen images were binarized with digital image processing technology, as shown in Figure 11.

Compared with the asphalt mixture containing single-grain aggregates (Figure 10), the asphalt mixture containing multi-grain aggregates showed a significantly reduced number of branching cracks. This means that the multi-grain aggregates effectively inhibited the growth of cracks, resulting in improved low-temperature cracking resistance. In addition, Figure 10 also reveals that greater particle size differences within the aggregates led to worse particle continuity and a larger number of branching cracks. This indicates that the continuity of the aggregates directly affected the crack propagation behavior of these asphalt mixtures.

The cracking resistance results at −20 °C are shown in Table 5. The aggregate enhancement effect is expressed as the ratio of the low-temperature cracking resistance of the asphalt mixture with aggregate to the low-temperature cracking resistance of the asphalt mortar (without aggregate).

According to Table 5, although the low-temperature cracking resistance of the asphalt mixture continuously increased (the maximum value was approximately 3.4 MPa), the ratio significantly decreased. This indicates that the aggregates enhanced the cracking resistance of the asphalt mixture, but the increase in the aggregate size difference resulted in the aggregate continuity decreasing, so the aggregate enhancement effect was limited.

Moreover, the poor continuity between aggregate particles increased the number of branching cracks in the specimens. However, an initial crack configuration is inevitable in asphalt pavement materials due to how they are formed. Therefore, crack configuration is an important factor that cannot be ignored when studying the particle reinforcement effect of aggregates.

### 4.2. The Influence of Crack Configurations

**Figure 12:**
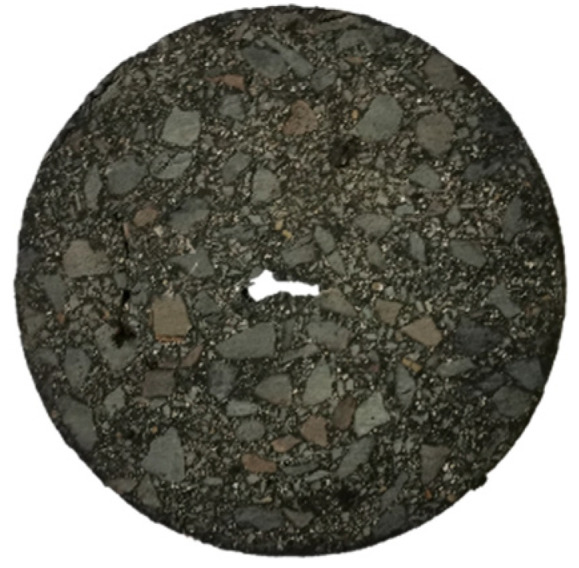
Pre-cracked asphalt mixture specimen with multi-grain aggregate (*D* = 2.36 mm and 4.75 mm).

The analysis mentioned above indicates that the asphalt mixture containing 2.36 mm and 4.75 mm aggregate particles did not have any branching cracks. Consequently, IDT tests were carried out with the asphalt mixture specimens containing aggregate particles with diameters of 2.36 mm and 4.74 mm. A waterjet was applied to form a pre-crack (with a crack length of *l* = 10 mm), as shown in Figure 12. 

**Figure 13:**
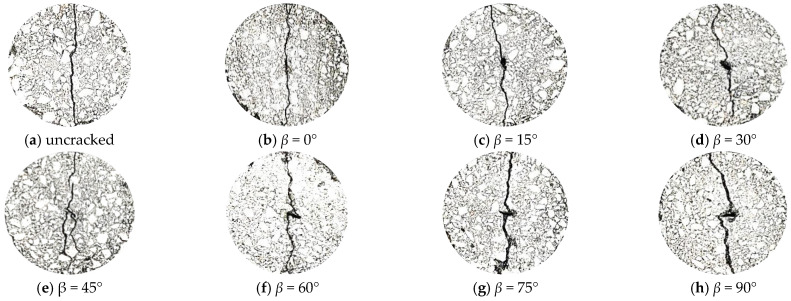
Results obtained under different initial crack deflection angles (*β*).

To directly observe internal crack propagation, images of the test specimens were binarized with digital image processing technology, as shown in Figure 13.

Compared with the uncracked asphalt mixture (Figure 13a), it is evident that when the crack deflection angle, *β* < 45°, the crack propagation deflection increases with increasing crack deflection angle. This indicates that the aggregate significantly interfered with crack propagation. Moreover, when *β* ≥ 45°, the crack propagation path was not significantly inhibited, indicating that the aggregate only weakly inhibited internal crack propagation in the asphalt mixture. The cracking resistance results from the IDT tests are shown in Table 6.

According to Table 6, when *β* < 45°, increasing the *β* value results in a gradual decline in the low-temperature cracking resistance of the asphalt mixture. This indicates a corresponding gradual decline in the aggregate enhancing effect. On the other hand, when *β* ≥ 45°, further increasing the *β* value causes the low-temperature cracking resistance of the pre-cracked asphalt mixture to increase. Overall, the low-temperature cracking resistance of the asphalt mixtures shows a trend of first decreasing and then increasing with increasing *β*, with a minimum value reached at *β* = 45°.

When *β* = 45°, the low-temperature cracking resistance of the asphalt mixtures is reduced by 34.95% compared with the non-pre-cracked sample, a more significant decline than that of the other specimens reported in Table 6. This specimen also has multiple branching cracks, indicating that there is a weak aggregate enhancement effect and no significant inhibition effect on crack propagation at this angle. In contrast, when *β* < 45° or *β* > 45°, branching cracks are successfully inhibited.

In general, these test results illustrate that aggregates enhance the low-temperature cracking resistance of asphalt mixtures. Their enhancing effect is affected by the continuity of the aggregates as well as by the crack configuration in the asphalt mixture. 

## 5. Crack Configurational Force

### 5.1. The Influence of Aggregates’ Relative Position on the Aggregate Enhancement Effect

In order to deeply reveal the interaction between crack configuration and aggregates, the XFEM and ABAQUS were used to establish a mesostructure model of the asphalt mixture. Based on the XFEM in ABAQUS 2022, the advancing front method was used to encrypt locally free meshes where the crack may pass through. And the crack was completely independent of the mesh, so there was no need to redivide the mesh during the crack propagation process. And the aggregates in the asphalt mixture were regarded as circular particles to avoid the influence of different configurations of the same-sized aggregate on the simulation results. And the bottom boundary was set as the fixed boundary condition.

**Figure 14:**
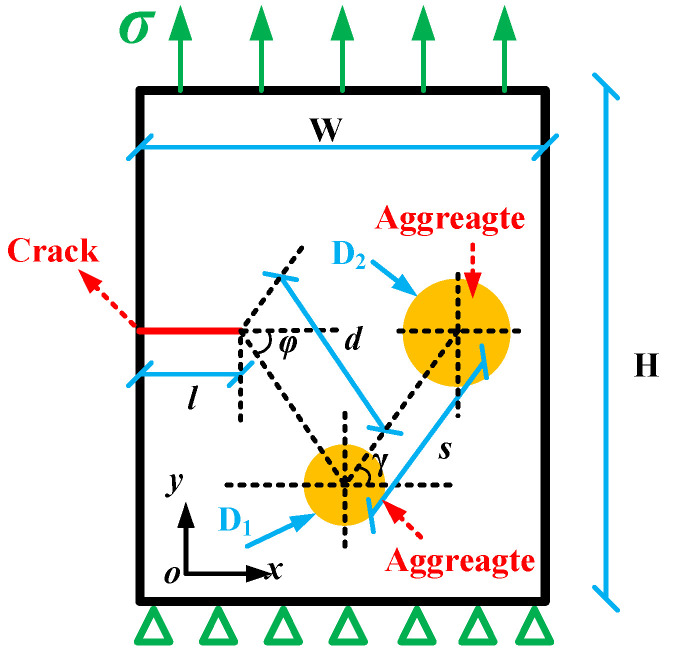
Asymmetric double aggregate model.

The relative position between aggregates is represented by the center distance, *s*, and the angle *γ*, which is the angle between the line connecting the center of the aggregate and the horizontal axis, *x*. And the numerical simulation takes *γ* = 0°, 15°, 30°, 45°, 60°, 75°, and 90°, respectively; *s* = 8 mm, *φ* = 45°, and *d* = 8 mm; and D_1_ = 2.36 mm and D_2_ = 4.75 mm, as shown in Figure 14.

Moreover, the stress, *σ*, at the upper boundary is equal to 4 MPa (see Figure 14), so the crack propagation condition (*C* ≥ *C*_th_) is always satisfied. The contact model between aggregates and the asphalt mortar adopted was the cohesive zone model (CZM), and the adhesive modeulus, *E* (GPa), and strength, *σ* (MPa), were set as the bonding surface failure conditions (see Table 7).

**Figure 15:**
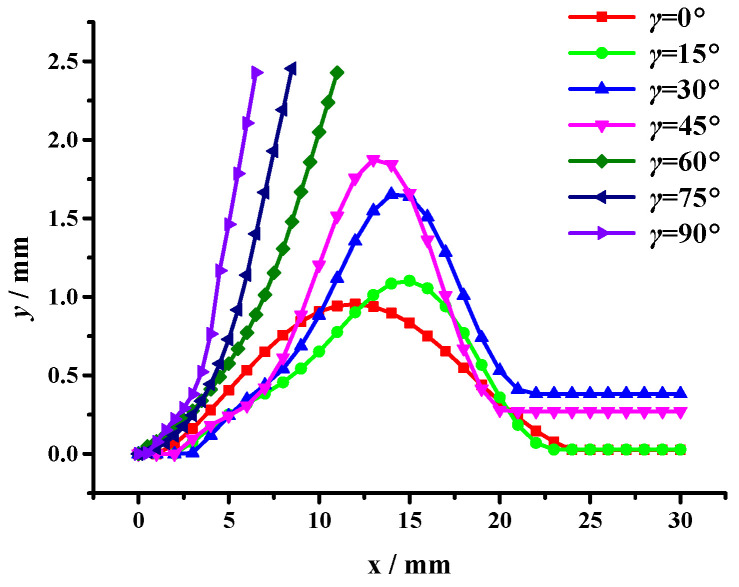
The crack propagation path under the asymmetric double aggregate interference effect.

According to the PHILSM field variables and taking the initial crack tip as the coordinate origin, changes in the crack tip coordinate (*x*, *y*) were extracted with crack propagation under the interference effect of aggregates with different sizes, i.e., the crack propagation path, as shown in Figure 15.

According to the crack propagation path results (see Figure 15), under a pure tensile load, when *γ* ≤ 45°, the crack is influenced by the interference of the aggregate and chooses to shift away from the aggregate. With the increase in *γ*, the double-aggregate interference effect on crack propagation becomes more obvious. Furthermore, when *γ* > 45°, the crack propagation path is deviated by the interference of the first aggregate until polymerization occurs with the second aggregate, and the crack propagation stops.

**Figure 16:**
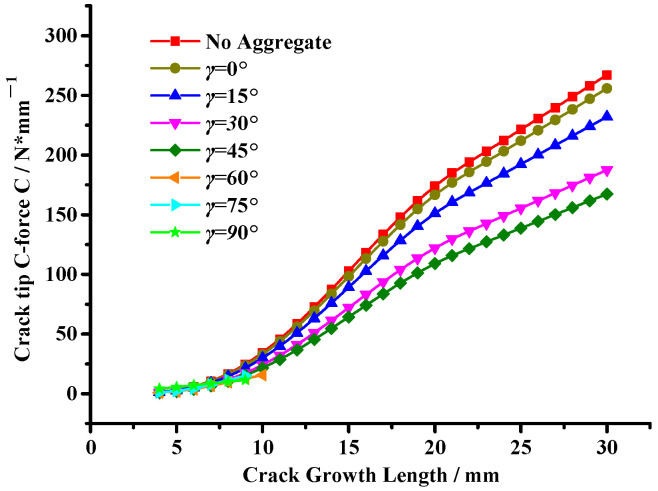
Variation in the crack tip’s configurational force under the asymmetric double aggregate interference effect.

Moreover, the crack tip’s configurational force change trends with the crack growth length are shown in Figure 16. And the reduction in the crack tip’s configurational force indicates a reduction in the driving force for crack propagation, meaning aggregates are beneficial to improve the cracking resistance of asphalt mixtures [32].

It can be seen from Figure 16 that the crack tip configurational force significantly decreases with an increase in the angle *γ*, meaning the aggregate inhibition effect on crack propagation is gradually enhanced. However, when the angle *γ* = 0°, there is little difference in the crack tip’s configurational force compared with the state without the aggregate interference effect, showing that the closer the relative position between aggregates, the smaller the aggregate interference effect on crack propagation.

In addition, combined with Figure 15, this illustrates that when *γ* > 45°, the crack propagation path deviates due to the interference of the first aggregate. And then the crack has a certain deflection angle, *β*, resulting in the second aggregate showing an inhibition effect on crack propagation, leading to a substantial decrease in the crack tip’s configurational force. This result shows that double aggregates show an inhibition effect on crack propagation, which effectively inhibits the growth of the crack tip’s configurational force and leads to the crack entering a stable state so that the asphalt mixture can obtain better cracking resistance.

Furthermore, this also shows that the crack deflection angle, *β*, affects the aggregate inhibition effect, so the aggregate interference effect on crack propagation under different crack deflection angles was studied as follows.

### 5.2. The Influence of the Crack Deflection Angle on the Aggregate Enhancement Effect

**Figure 17:**
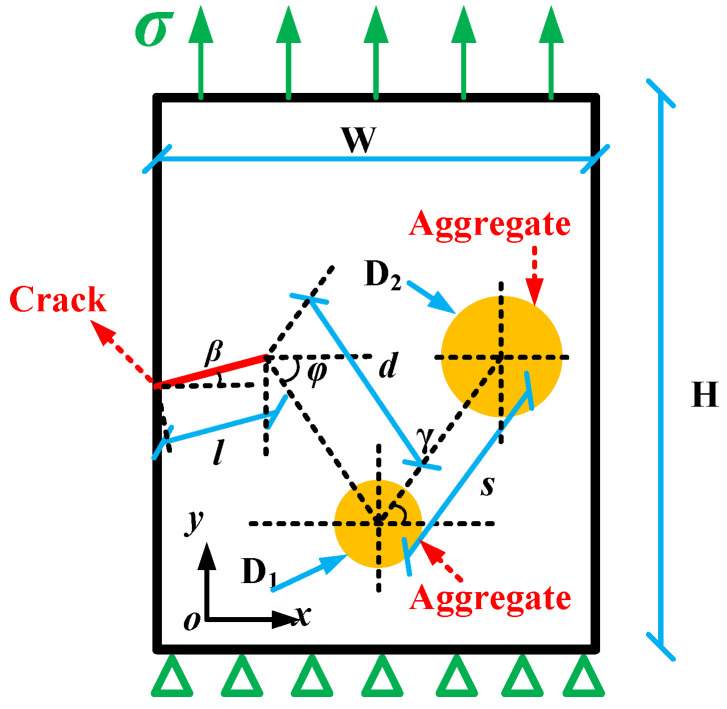
The asymmetric double-aggregate interference model under different crack deflection angles.

According to the results mentioned above, when angle *γ* > 45°, the aggregate has a crack inhibition effect on crack propagation. Therefore, the numerical simulation of crack propagation takes *γ* = 45°, *s* = 8 mm, *φ* = 45°, *d* = 8 mm; the crack deflection angle *β* takes values of 0°, 15°, 30°, 45°, 60°, and 75°, respectively; and D_1_ = 2.36 mm and D_2_ = 4.75 mm, as shown in Figure 17. And the boundary of the model is consistent with the former model mentioned above.

**Figure 18:**
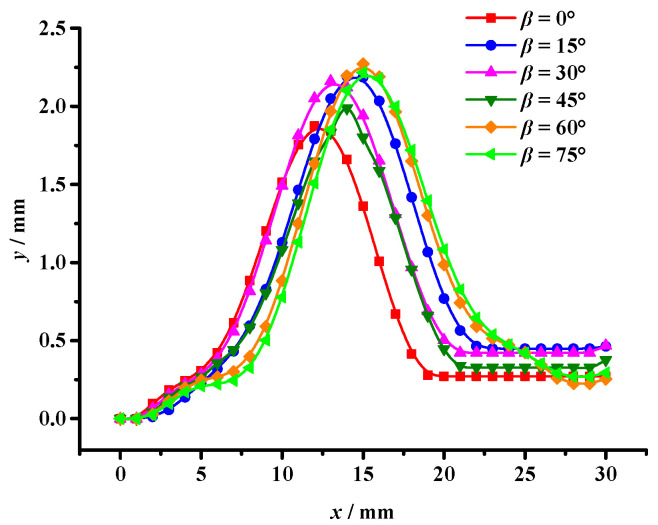
The crack propagation path under different crack deflection angles.

According to the PHILSM field variables, the crack propagation path can be extracted, as shown in Figure 18.

It can be seen from Figure 18 that although the crack propagates in the form of an I-mode crack after cack initiation, the crack propagation path deviates to varying degrees under different crack deflection angles, *β*. Furthermore, the initial deviation in the crack in the y-axis direction gradually decreases with increasing values of *β*, which indicates that the first aggregate’s interference effect on crack propagation gradually weakens.

**Figure 19:**
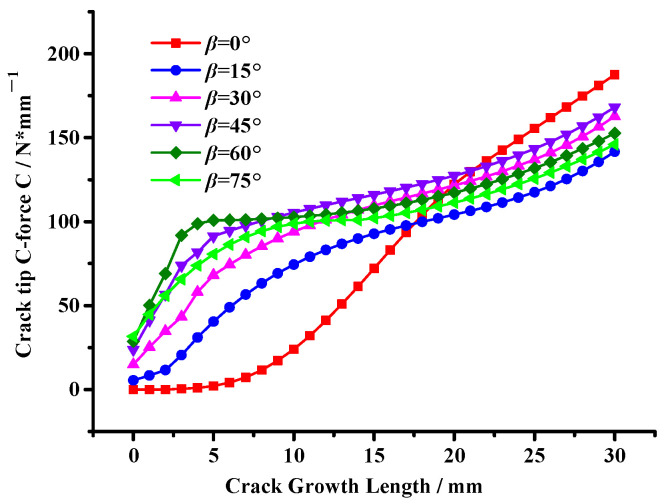
Variation in the crack tip’s configurational force under different crack deflection angles.

Figure 19 shows that as the crack deflection angle, *β*, increases, the crack tip’s configurational force gradually increases. This further illustrates that the first aggregate’s interference effect on crack propagation gradually weakens, causing the crack to tend towards an unstable state. Meanwhile, there are significant differences in the growth trend of the crack tip’s configurational force, i.e., the crack tip’s configurational force rapidly increases when *β* = 0°, while there are different degrees of slowdown in increasing trends when *β* ≠ 0°, meaning the second aggregate shows different degrees of inhibition on crack propagation.

Moreover, by comparing the crack tip’s configurational force when *β* ≠ 0° (see Figure 19), it can be found that the crack tip’s configurational force is the largest when *β* = 45°, indicating that the aggregate inhibitory effect on crack propagation is weak, leading to the crack tending to be unstable and prone to propagation, resulting in the formation of macroscopic cracks, making the asphalt mixture prone to damage, which is consistent with the experimental results mentioned above.

## 6. Results and Discussion

In this study, a static pressure method and a waterjet were used to form the asphalt mixture with pre-cracks. Moreover, combined with the IDT test and the XFEM, the mesostructure model of the asphalt mixture was established to study the enhancing effect of aggregates on the low-temperature cracking resistance of the asphalt mixture and the influence of crack configuration. The main conclusions are as follows:

(1) An increase in aggregate particle size decreases the crack configurational force, contributing to enhancing the low-temperature cracking resistance of asphalt mixtures. Although aggregates with larger particle sizes have a stronger inhibitory effect on crack propagation, this inhibitory effect diminishes with increasing aggregate particle size.
(2) Compared with single-grain aggregates, multi-grain aggregates significantly reduce the number of internal branching cracks, showing that multi-grain aggregates effectively inhibit crack propagation. However, an excessive disparity in particle sizes compromises particle continuity, leading to the formation of more branching cracks.
(3) The double-aggregate interference effect on crack propagation shows an inhibition effect; the growth of the crack tip’s configurational force can be effectively inhibited, and the crack tends to a stable state so that the asphalt mixture can obtain better crack resistance. In addition, the inhibit effect is influenced by the crack deflection angle, *β*, limiting the aggregate enhancement effect on the cracking resistance of the asphalt mixture.

These results lay the foundation for in-depth research on crack propagation evolution behavior and crack resistance mechanisms and provide a theoretical basis for optimizing the composition of asphalt mixtures to improve their durability and mechanical properties.

## Figures and Tables

**Table 1:** Asphalt mixture gradation.

Sieve Size (mm)	<0.075	0.075~0.15	0.15~0.3	0.3~0.6	0.6~1.18	1.18~2.36
AC-13 sieve residual percentage (%)	6	5	4.5	8.5	8	10.5
Asphalt mortar sieve residual percentage (%)	14.1	11.8	10.6	20	18.8	24.71

**Table 2:** Coarse aggregate mass ratio.

Sieve Size (mm)	2.36~4.75	4.75~9.5	9.5~13.2	13.2~16
AC-13 sieve residual percentage (%)	20	21.5	13.5	2.5
Mass ratio	0.470588	0.505882	0.317647	0.058824

**Table 3:** The mechanical properties of AC-13-grade asphalt mixture at −20 °C.

Materials	The Calculation Mechanical Parameters	Values
Aggregate	Elastic Modulus, *E*	55.5 GPa
	Tensile Strength, *σ*	27.6 MPa
	Poisson’s Ratio, *ν*	0.25
Asphalt mixture	Tensile Strength, *σ*	3.55 MPa
Asphalt Mortar	Dynamic Modulus, *E*	0.832 GPa
	Poisson’s Ratio, *ν*	0.23
	Tensile Strength, *σ*	0.73 MPa
	Fracture Energy	275 J/m^2^
Contact Layer between Aggregate and Asphalt	Adhesive Modulus, *E*	0.596 GPa
	Adhesive Strength, *σ*	0.73 MPa

**Table 4:** Enhancement effect of different aggregate sizes.

Test Specimen		Without Pre-Cracks		With Pre-Cracks (*l* = 10 mm, *β* = 45°)	
		Crack Resistance (MPa)	Enhancement Effect	Crack Resistance (MPa)	Enhancement Effect
Asphalt mortar		1.889	—	1.186	—
Aggregate size (mm)	2.36	2.456	1.30	1.633	1.38
	4.75	2.987	1.58	2.055	1.73
	9.5	3.366	1.78	2.795	2.36
	13.2	3.416	1.81	3.048	2.57

**Table 5:** Low-temperature cracking resistance test results.

Test Specimen		Cracking Resistance (MPa)	Enhancement Effect	Ratio
Asphalt Mortar		1.889		
Asphalt mixture with the multi-grain aggregate	2.36 mm/2.36 mm	2.456	1.3	--
	2.36 mm/4.75 mm	3.067	1.62	0.32
	2.36 mm/9.5 mm	3.413	1.81	0.19
	2.36 mm/13.2 mm	3.437	1.82	0.01

**Table 6:** Cracking resistance results obtained under different initial crack deflection angles.

Crack Deflection Angle	Cracking Resistance (MPa)	Change in Cracking Resistance Compared with Uncracked Specimen
Without pre-cracks	3.067	—
*β* = 0°	2.988	−2.58%
*β* = 15°	2.612	−14.83%
*β* = 30°	2.499	−18.52%
*β* = 45°	1.995	−34.95%
*β* = 60°	2.321	−24.31%
*β* = 75°	2.478	−19.20%
*β* = 90°	2.571	−16.18%

**Table 7:** The model parameters of the XFEM.

Materials	Model Parameters	Value
Asphalt Mixture	Width, W	30 mm
	Length, H	40 mm
Crack	Crack length, *l*	2 mm
Aggregates	Particle size	2.36 mm, 4.75 mm
	Distance, *d*	8 mm
	Angle, *φ*	45°

## Data Availability

The original contributions presented in the study are included in the article, further inquiries can be directed to the corresponding author.
